# 1-(2-aminophenyl)-1H-1,2,3-triazole-4-carboxylic acid: activity against Gram-positive and Gram-negative pathogens including *Vibrio cholerae*

**DOI:** 10.1098/rsos.170684

**Published:** 2017-10-18

**Authors:** Krishnendu Maji, Debasish Haldar

**Affiliations:** Department of Chemical Sciences, Indian Institute of Science Education and Research Kolkata, Mohanpur, West Bengal 741246, India

**Keywords:** ε-amino acid, click chemistry, antimicrobial, triazole

## Abstract

We report a new synthetic aromatic ε-amino acid containing a triazole moiety with antimicrobial potential against Gram-positive, Gram-negative and pathogenic bacteria including *Vibrio cholerae*. Structure–property relationship studies revealed that all the functional groups are essential to enhance the antimicrobial activity. The 1-(2-aminophenyl)-1H-1,2,3-triazole-4-carboxylic acid was synthesized by click chemistry. From X-ray crystallography, the amino acid adopts a kink-like structure where the phenyl and triazole rings are perpendicular to each other and the amine and acid groups maintain an angle of 60°. The agar diffusion test shows that the amino acid has significant antibacterial activity. The liquid culture test exhibits that the minimum inhibitory concentration (MIC) value for *Bacillus subtilis* and *Vibrio cholerae* is 59.5 µg ml^−1^. FE-SEM experiments were performed to study the morphological changes of bacterial shape after treatment with compound **1**. The antimicrobial activity of the amino acid was further studied by DNA binding and degradation study, protein binding, dye-binding assay and morphological analysis. Moreover, the amino acid does not have any harmful effect on eukaryotes.

## 1. Introduction

Infectious diseases are responsible for a large number of human deaths worldwide. However, the rise in the bacterial resistance to popular antibiotics poses a serious threat to public health [1]. So, new drugs with improved efficacy are desired. The medicinal chemists have shorted out that the development of more powerful antibiotics is not the only solution to this problem [2]. Rather, developing new antibiotics that act through mechanisms different from those of existing drugs will be more effective [3]. In this regard, it is very important to develop novel, efficient antimicrobial agents which have different functionalities from common antibiotics and which may have clinically unexploited modes of action [4]. Antimicrobial peptides are ubiquitous in nature, found in almost every multicellular organism [5]. They have attracted considerable attention because of their broad-spectrum activity and relatively low bacterial resistance [6]. The θ-defensins RTD-1 and RTD-2 from Rhesus macaque [7], the protegrin-1 (PG-1) from porcine leucocytes [8], polyphemusin-1 (PM-1) from horseshoe crab haemocytes [9] and BTD-2 from baboon [10] are interesting as natural antimicrobial peptides with a cyclic backbone. Robinson and co-workers have used PG-1 as a starting point for the development of a potent and specific lead compound with nanomolar activity against *Pseudomonas aeruginosa* [11]. Chemical modification or substitution at single (or multiple) sites of these natural amino acids are emerging strategies to modulate antimicrobial activity of such host defence peptides [12]. On the other hand, information gleaned from the study of these peptides has been used in the design of antimicrobial agents using synthetic building blocks [13]. Particularly, conformationally constrained new amino acids with desired functionalities are very much important because of their immunity towards enzymatic degradation. In this context, we are developing antimicrobial amino acid building blocks [14]. Here, we have incorporated a triazole moiety [15] in an amino acid backbone. Triazole with an N-N = N entity is promising as a versatile bioactive heterocycle [16]. Triazoles have a wide presence in many synthetic drugs of several infective diseases such as tuberculosis [17], malaria [18], inflammation [19], fungal and bacterial infection [20].

We are developing designer amino acids, peptides and analogues with desired functionalities [21]. Recently, we have described the synthesis of 2-acetyl amino-3-[4-(2-amino-5-sulfo-phenylazo)-phenyl]-propionic acid starting from phenylalanine, as a hen egg white lysozyme (HEWL) amyloid inhibitor [22]. Here, we report the synthesis and antimicrobial activities of an aromatic ε-amino acid containing a triazole moiety. 1-(2-aminophenyl)-1H-1,2,3-triazole-4-carboxylic acid was synthesized by click chemistry using copper(II) sulphate pentahydrate and sodium ascorbate as catalyst [23]. The X-ray crystallography sheds some light on the atomic-level structure of the reported ε-amino acid. Based on the agar diffusion test, liquid culture test, DNA binding and degradation study, dye-binding assay and morphological analysis, we conclude that the new amino acid acts as a broad-spectrum antibiotic against Gram-positive, Gram-negative and pathogenic bacteria but does not have any harmful effect on eukaryotes.

## 2. Material and methods

### 2.1. General

All chemicals were purchased from Sigma chemicals.

### 2.2. Amino acid synthesis

#### 2.2.1. Preparation of 2-pthalimidonitrobenzene

For the preparation, 14.18 g of phthalic anhydride (100 mmol) and 13.18 g of 2-nitroaniline were mixed together and heated at 215°C for 2 h and the reaction mixture was allowed to cool at room temperature, and 50 ml of glacial acetic acid was added into it. The resulting solution was refluxed for another half an hour to dissolve the unreacted phthalimide. After 5 min, the reaction mixture was slowly cooled and the resulting mixture was filtered and the precipitate was washed with diethyl ether until a yellow-coloured crystalline solid appeared (19.16 g, 72%): mp 195–196°C. ^1^H NMR (DMSO-*d_6_*, 400 MHz, *δ* in ppm): 8.25–8.23 [d, *J* = 8 Hz, 1H, Aromatic proton], 8.05–8.02 [m, 2H, Aromatic protons], 7.98–7.95 [ m, 3H, Aromatic protons], 7.80–7.76 [m, 2H, Aromatic protons]. ^13^C NMR (DMSO-*d_6_*, 100 MHz, *δ* in ppm): 166.1, 145.5, 135.3, 134.7, 131.2, 130.3, 125.4, 124.7, 123.9.

#### 2.2.2. Preparation of 2-phthalimidoaniline (2)

To a solution of 10.73 g of 2-pthalimidonitrobenzene, 200 ml of acetone, 32 ml of glacial acetic acid, 32 ml of water and 26.81 g of iron powder (480.0 mmol) were added. The reaction mixture was heated to reflux. After 8 h, the reaction mixture was cooled and filtered through a pad of Celite. The filtrate was concentrated under vacuum. From the filtrate the compound was obtained as a yellow-coloured solid after column chromatography using ethyl acetate and hexane as the mobile phase and silica as the solid phase (7.04 g. 74%): ^1^H NMR (CDCl_3_, 400 MHz, *δ* in ppm): 7.93–7.91 [m, 2H, Aromatic protons], 7.78–7.74 [m, 2H, Aromatic protons], 7.25–7.20 [m, 1H, Aromatic proton], 7.1–7.07 [m, 1H, Aromatic proton], 6.88–6.84 [t, *J* = 8 Hz, 2H, Aromatic proton], 4.32 [br, 2H, NH_2_ protons] ^13^C NMR (CDCl_3_, 100 MHz, *δ* in ppm): 167.8, 143.1, 134.8, 132.4, 130.0, 129.6, 124.2, 119.7, 118.4.

#### 2.2.3. Preparation of 2-phthalimidophenyl azide (3)

For this preparation, 5.52 g of **2** (25 mmol) was suspended in 160 ml of glacial acetic acid and 160 ml of water. The mixture was cooled to 0°C, and 2.42 g of NaNO_2_ (35.0 mmol) was added in one portion. After 3 h, the remaining undissolved starting **2** was isolated by filtration. To the filtrate, 2.44 g of NaN_3_ (37.5 mmol) was added slowly and during the addition a small portion of ethyl ether was added to stop excessive foaming. After half an hour, the reaction mixture was filtered to get **3** as a light yellow solid (6.37 g, 99%): ^1^H NMR (CDCl_3_, 400 MHz, *δ* in ppm): 7.95–7.93 [m, 2H, Aromatic protons], 7.79–7.78 [m, 2H, Aromatic protons], 7.51–7.48 [m, 1H, Aromatic protons], 7.31–7.24 [m, 3H, Aromatic protons]. ^13^C NMR (CDCl_3_, 100 MHz, *δ* in ppm): 167.4, 138.3, 134.8, 132.4, 131.1, 125.8, 124.3, 123.0, 119.8.

#### 2.2.4. Synthesis of 2-aminophenyl azide (4)

For this synthesis, 5.55 g of 2-phthalimidophenyl azide (21.0 mmol) was suspended in 14 ml of methanol. To this slurry, 1.5 ml of hydrazine hydrate (31.5 mmol) was added in one portion. The resulting reaction mixture was stirred rapidly. After 20 min, the mixture was very thick and into it another 10 ml of methanol was added, and stirring was continued for another 1 h. To the resulting paste, 40 ml of 1(M) aqueous solution of NaOH was added. The mixture was diluted with 30 ml of water and 60 ml of DCM. The resulting two phases were separated and the aqueous extract was separated with another 2 × 30 ml of DCM. The combined organic phase was washed with 1 × 30 ml of distilled water and 1 × 30 ml of brine. The resulting organic solution was dried over Na_2_SO_4_ and the homogeneous mixture was concentrated under vacuum. The compound was purified by column chromatography using ethyl acetate and hexane as the mobile phase and silica gel as the solid phase (1.76 g. 62%): ^1^H NMR (DMSO-*d_6_*, 400 MHz, *δ* in ppm): ^1^H NMR (DMSO-*d_6_*, 400 MHz, *δ* in ppm): 7.02–6.99 [m, 1H, Aromatic proton], 6.90–6.87 [m, 1H, Aromatic proton], 6.65–6.60 [m, 2H, Aromatic protons], 4.95 [br, 2H, NH_2_ proton]. ^13^C NMR (DMSO-*d_6_*, 100 MHz, *δ* in ppm): 139.9, 125.7, 123.0, 118.6, 116.7, 115.0.

#### 2.2.5. Synthesis of 1-(2-aminophenyl)-1H-1,2,3-triazole-4-carboxylic acid (1)

To a 1:1 mixture of 2-aminophenyl azide (1.34 g, 10 mmol) and propiolic acid (0.7 g, 10 mmol) in a 1:1 ethanol and water solvent in a 250 ml round bottom flask, 0.2 mmol sodium ascorbate and 0.05 mmol CuSO_4_ were added, and this was covered with an aluminium foil followed by stirring at room temperature for 12 h. The resulting mixture was concentrated under vacuum, and amino acid was obtained after separating the mixture by column chromatography using ethyl acetate and hexane as the mobile phase and silica gel as the solid phase (1.59 g, 78%): ^1^H NMR (DMSO-*d_6_*, 400 MHz, *δ* in ppm): 8.92 [s, 1H, Triazole CH proton], 7.24–7.21 [m, 2H, Aromatic protons], 6.92–6.90 [d, *J* = 8 Hz, 1H, Aromatic proton], 6.69–6.66 [t, *J* = 6 Hz, 1H, Aromatic proton], 5.40 [br, 2H, NH_2_ protons]. ^13^C NMR (DMSO-*d_6_*, 100 MHz, *δ* in ppm): 161.7, 142.9, 139.9, 130.6, 130.08, 126.2, 121.4, 116.6, 115.9.

#### 2.2.6. NMR experiments

All NMR studies were carried out on a Jeol 400 MHz spectrometer at 278 K. Compound concentrations were in the range of 1–10 mM in CDCl_3_ and (CD_3_)_2_SO.

#### 2.2.7. FT-IR spectroscopy

All reported solid-state FT-IR spectra were obtained with a Perkin Elmer Spectrum RX1 spectrophotometer with the KBr disc technique.

#### 2.2.8. UV/vis spectroscopy

Absorption spectra were recorded on a Perkin Elmer spectrophotometer.

#### 2.2.9. Field emission scanning electron microscopy

Morphological changes of bacterial shape after treatment with compound **1** were investigated using field emission-scanning electron microscopy (FE-SEM). Aliquots of 10 ml of the liquid culture of each *E. coli* (*E. coli* XL10), *Bacillus subtilis* and *Vibrio cholerae* O395 bacteria were incubated and allowed to grow for 8 h in the presence of compound **1**; 5 ml aliquots from each culture were centrifuged at 5000 r.p.m. for 5 min. The supernatants were discarded. The pellets were washed with water two times and then with 1% PBS buffer two times. The resultant solutions for each set of bacteria were incubated at RT for 30 min and then at 4°C overnight. The suspensions were washed with PBS buffer three times. Then the pellets were collected by centrifugation. Then each sample was dehydrated using 200 µl of different grades of ethanol like 30%, 50%, 70%, 80%, 90% and 100%, respectively. The samples were incubated for 1 h, and 5 µl from each set for each sample was drop cast on a glass slide and dried under vacuum. Similarly, samples were prepared for the control experiment, that is bacteria in the absence of compound **1**. The materials were gold-coated, and the micrographs were taken in an FE-SEM apparatus (Jeol Scanning Microscope-JSM-6700F).

### 2.3. Single crystal X-ray diffraction study

Crystallographic data of compound **1**: C_9_H_8_N_4_O_2_, Mw = 236.24, monoclinic, space group P 1 21/n 1, *a* = 10.7631(4), *b* = 4.5960(2), *c* = 22.5078(9) Å, *α* = 90°, *β* = 94.127(4), *γ* = 90°, *V* = 13637.9(9) Å^3^, *Z* = 4, dm = 1.413 Mg m^−3^, *T* = 100 K, R1 0.0422 and wR2 0.1082 for 1952 data with *I* > 2*σ*(*I*). Intensity data were collected with MoKα radiation using a Bruker APEX-2 CCD diffractometer. Data were processed using the Bruker SAINT package and the structure solution and refinement procedures were performed using SHELX97. The data for compound **1** have been deposited at the Cambridge Crystallographic Data Centre with reference number CCDC 1543560.

### 2.4. Agar diffusion test

A small amount of bacteria (*E. coli* XL10 and *Bacillus subtilis*) culture was spread over the whole agar plate that contained some tiny pores; inside the pores, the aqueous solution of the test compound and the reference were placed separately and the plate was left for incubation for 10 h at a temperature of 37°C; the zone of inhibition has been calculated. For the agar diffusion test on *Bacillus subtilis,* 150 µl of an aqueous solution of compound **1** with concentration 20 mg ml^−1^ and 20 µl of an aqueous solution of kanamycin with a concentration of 50 mg ml^−1^ were used for the test and the reference, respectively. A 35 µl solution of compound **1** with a concentration of 20 mg ml^−1^ and a 14 µl aqueous solution of kanamycin with a concentration of 50 mg ml^−1^ were used for the agar diffusion test of *E. coli*.

### 2.5. MIC test

Bacteria are allowed to grow in different identical sets of liquid media (LB media) containing variable concentrations of compound **1**. The growth of bacteria for each set is measured by recording the optical density of the solution at 600 nm after each one hour time interval until the bacterial growth has become saturated. Then the optical density versus time is plotted for each set to get the growth curve for all the bacteria. From that growth curve, the MIC value has been calculated. In a conical tube containing 10 ml of LB media, *E. coli* bacteria (*E. coli* XL10) are incubated for 8 h at 37°C and the growth of *E. coli* are measured by recording the optical density of the solution at 600 nm. Then 200 µl of the bacterial suspension is added into each conical tube containing the test compound with final concentrations of 34, 42.5, 51, 59.5, 68, 76.5 and 85 µg ml^−1^ in 10 ml of LB media, respectively. The parallel control experiment is done by adding the same 200 µl of the bacterial suspension to each conical tube containing 10 ml of LB media alone and 30 µg ml^−1^ kanamycin in 10 ml of LB media, respectively. The growth of bacteria for each conical tube is measured by recording the optical density of the solution at 600 nm after each one hour time interval until the bacterial growth becomes saturated. The optical density versus time are plotted together. The black sigmoidal growth curve is for only culture that is in the absence of any test compound.

### 2.6. DNA-binding assay

A 10 ml aliquot of the liquid culture of *E. coli* bacteria was incubated at 37°C and allowed to grow for 8 h. Then 1.5 ml of culture was centrifuged under 5000 r.p.m. and the supernatant was discarded. The pellets were resuspended in 567 µl of TE (Tris-EDTA) buffer followed by addition of 30 µl of 10% SDS and 3 µl of 20 mg ml^−1^ proteinase K in order to make a final concentration of 100 µg ml^−1^ proteinase K in 0.5% SDS. The resultant solution was mixed thoroughly and allowed to incubate for 1 h at 37°C. Then 100 µl of 5 M NaCl was added to the solution with thorough mixing followed by the addition of 80 µl of CTAB (cetyl trimethyl ammoniumbromide)/NaCl solution. This resultant mixture was mixed thoroughly and incubated for 10 min at 65°C. These two steps are very vital because the addition of 100 µl of 5 M NaCl keeps the salt concentration above 0.5 M. At this salt concentration, cell wall debris, denatured protein and polysaccharides formed a complex with CTAB, while the nucleic acid remained unaffected. To extract CTAB-cell wall debris/denatured protein and polysaccharides complex, 0.7 ml of chloroform/isoamyl alcohol was added and the solution was mixed thoroughly and centrifuged at 5000 r.p.m. for 5 min. A white interface containing the CTAB-cell wall debris/denatured protein and polysaccharides complex was found to appear; this interface was discarded and the viscous supernatant was transferred to a microcentrifuge tube containing an equal volume of phenol/chloroform/isoamyl alcohol. The resultant solution was centrifuged under 5000 r.p.m. for 5 min. The supernatant was transferred to a fresh tube and 0.6 volume of isopropanol was added to precipitate the nucleic acid. The tube was shaken gently till the white nucleic acid precipitate appeared. The nucleic acid precipitate was dissolved in 70% ethanol. Similarly, the nucleic acid was collected from compound **1**-treated *E. coli*.

### 2.7. Protein isolation

A 10 ml aliquot of the liquid culture of only *E. coli* bacteria and compound **1**-treated *E. coli* bacteria were incubated separately and allowed to grow for several hours; 1.5 ml of the control culture and 4.5 ml of the test culture were centrifuged at 5000 r.p.m. for 5 min. The supernatant was discarded. Then each of the pellets was suspended in 200 µl of Tris-NaCl buffer (10 mM Tris and 50 mM NaCl) followed by addition of 4× Sample Loading Buffer (SLB compositions). The solutions were boiled and sonicated and again boiled. Now, on a Whatman filter paper 15 squares, each of 1.5 × 1.5 cm, were drawn. During the experiment, it was ensured that the filter paper was not touched with any surface from which it could absorb any protein. A standard protein solution of a known amount (ranging from 1 µg to 4 µg) was drop cast on the centre of each small square in triplicate. Similarly, the test sample and the control sample were drop cast in triplicate. The paper was allowed to air-dry and then washed with methanol to remove the interfering substances during dye binding for 10–20 min, and allowed to dry again. The paper was stained in 200 ml of 0.5% Coomassie brilliant blue G in 7% acetic acid for 45 min at room temperature with gentle shaking. Then the destaining of the filter paper was done in 7% acetic acid until the background became adequately reduced. The filter paper was air-dried. After complete drying, each small square was cut and put into a 2 ml microtube; 1 ml of the extraction buffer (66% methanol, 33% water, 1% ammonium hydroxide) was added and the tube was shaken thoroughly. This buffer extracts the proteins from the paper. The optical density of the solution was recorded in a spectrophotometer at a 610 nm wavelength. The optical densities of the sample were plotted against the amount of protein (in µg). From the plot, we can quantify the amount of protein in the control sample and the test sample. Equal amounts of protein from the control sample and the test sample are allowed to run by Protein-gel electrophoresis under a 15 mA current for 90 min. The gel is stained in 200 ml of 0.5% Coomassie brilliant blue G in 7% acetic acid for 45 min at room temperature with gentle shaking followed by destaining in 7% acetic acid.

## 3. Results and discussion

**Scheme 1.**
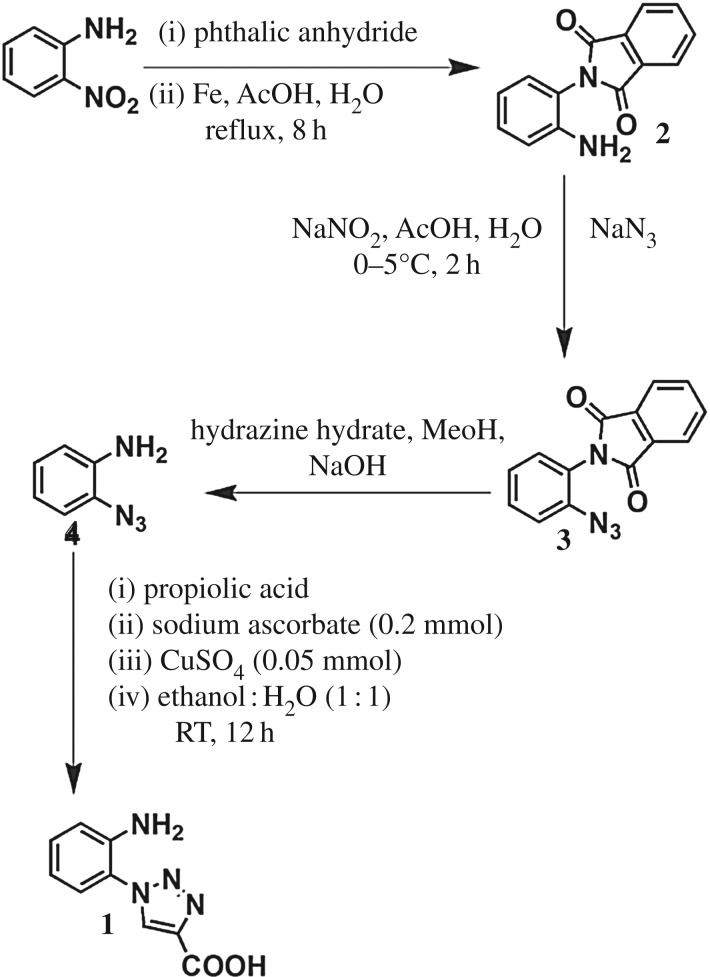
The synthesis of 1-(2-aminophenyl)-1H-1,2,3-triazole-4-carboxylic acid **1**.

For compound **1**, we have incorporated a triazole moiety to enhance the antimicrobial activity [24]. The 1-(2-aminophenyl)-1H-1,2,3-triazole-4-carboxylic acid was synthesized by click chemistry (scheme 1) between 2-aminophenyl azide and propiolic acid using copper(II) sulphate pentahydrate and sodium ascorbate as catalyst [23]. Compound **1** was purified and characterized by ^1^H-NMR,^13^C-NMR and FT-IR analysis. X-ray crystallography confirmed the formation of compound **1**.

To investigate the conformational preference of compound **1**, solid-state FT-IR spectroscopy was performed. From FT-IR spectra, the bands at 3427 cm^−1^ indicate that all NH groups are not hydrogen-bonded (electronic supplementary material, figure 1). The broad peak around 3300 cm^−1^ also suggests that the compound has extensively hydrogen-bonded network structures.

**Figure 1.**
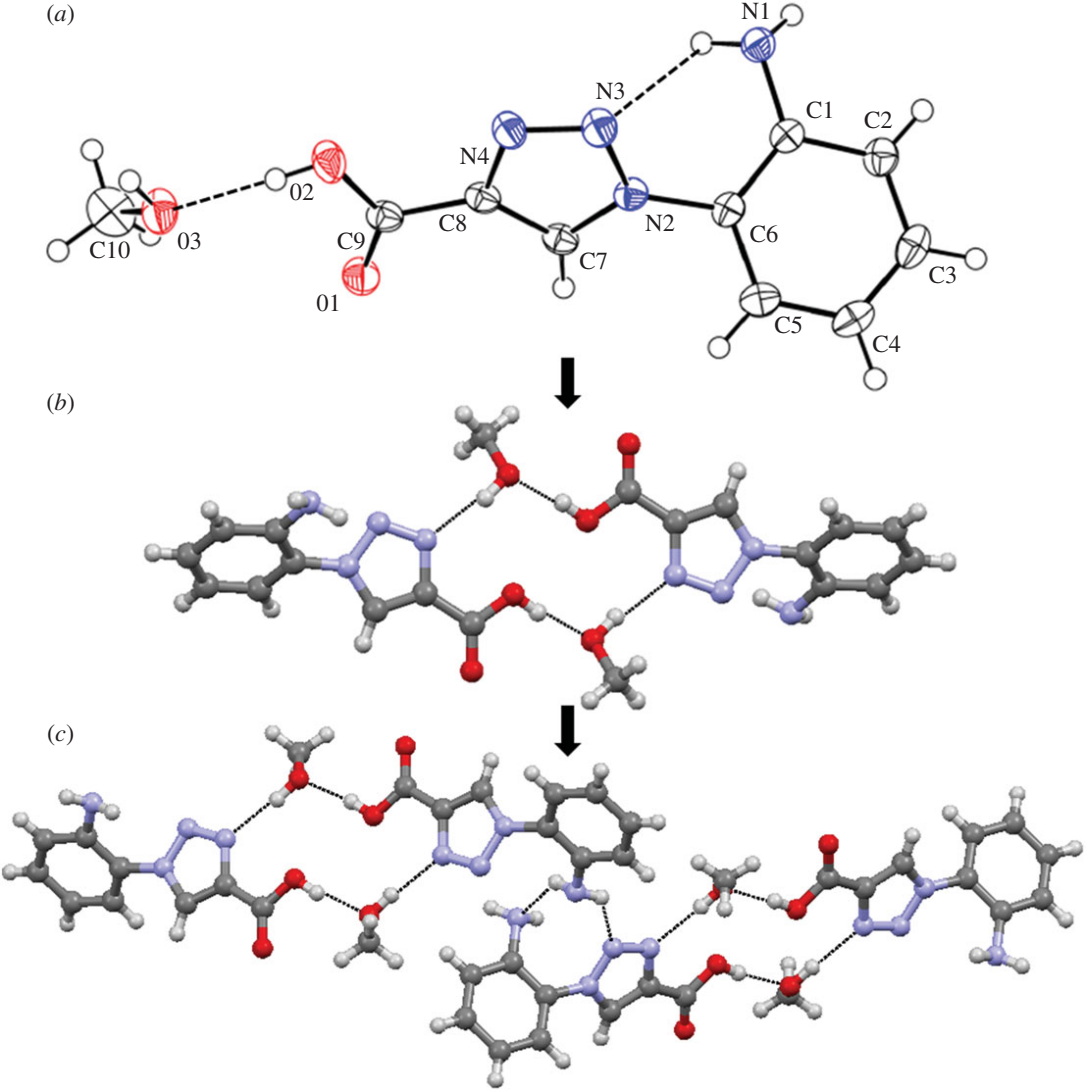
(*a*) The ORTEP diagram of compound **1**. Ellipsoids are drawn at the 50% probability level. Hydrogen bonds are shown as dotted lines. (*b*) Solvent-mediated dimer of compound **1**. (*c*) The complex sheet-like structure of compound **1**.

Colourless monoclinic crystals of compound **1** were obtained from a methanol–water solution by slow evaporation. The X-ray crystallography shows that the asymmetric unit contains one molecule of compound **1** and a methanol molecule. The ORTEP diagram (figure 1*a*) shows that compound **1** adopts a kink-like conformation. The phenyl and triazole rings are perpendicular to each other. Moreover, the amine and acid groups maintain an angle of 60° like *o*-aminobenzoic acid moiety. There is an intramolecular N-H…N hydrogen bond between amine NH and triazole N. There exists another O–H…O hydrogen bond between acid OH and methanol oxygen (figure 1*a*).

From the packing diagram, it can be seen that the individual amino acid building blocks are themselves regularly interlinked through methanol-mediated intermolecular hydrogen-bonding interactions, O3–H3A…N4, and thereby form a 14-member ring-like structure (figure 1*b*) along the crystallographic *a* direction. In higher-order packing, compound **1** forms a complex sheet-like structure through multiple intermolecular hydrogen bonds and π–π stacking interaction (minimum C-N distance 3.72 Å) (figure 1*c*) along the crystallographic *c* direction. The hydrogen-bonding parameters are listed in table 1.

**Table 1.** Hydrogen-bonding parameters of compound **1**^a^ (symmetry equivalent).

D–H…A	D…H (Å)	H…A (Å)	D…A (Å)	D–H…A (°)
N1–H1A…N3	0.86	2.56	3.147(2)	126^a^
N1–H1B…N2	0.86	2.54	2.890(2)	106
N1–H1B…N3	0.86	2.52	2.935(2)	111
N1–H1B…N1	0.86	2.46	3.115(2)	134^a^
O2–H2…O3	0.82	1.74	2.552(2)	169
O3–H3A…O2	0.88	2.59	3.087(2)	117^b^
O3–H3A…N4	0.88	1.94	2.808(9)	169^b^

^a^*a *= 3/2 − *x*, −1/2 + *y*, 3/2 − *z*.

^b^*b* = 2 − *x*, 1 − *y*, 1 − *z*.

The agar diffusion test is a well-known procedure to check the bacterial sensitivity because of its simplicity, low cost, the ability to test enormous numbers of microorganisms and antimicrobial agents together, and ease of interpretation of the test results [25]. Here, we used compound **1** as the test compound and kanamycin as the reference. The result shows that the trizole-based compound **1** has significant antibacterial activity against *E. coli* and *Bacillus subtilis* (electronic supplementary material, figure 2).

**Figure 2.**
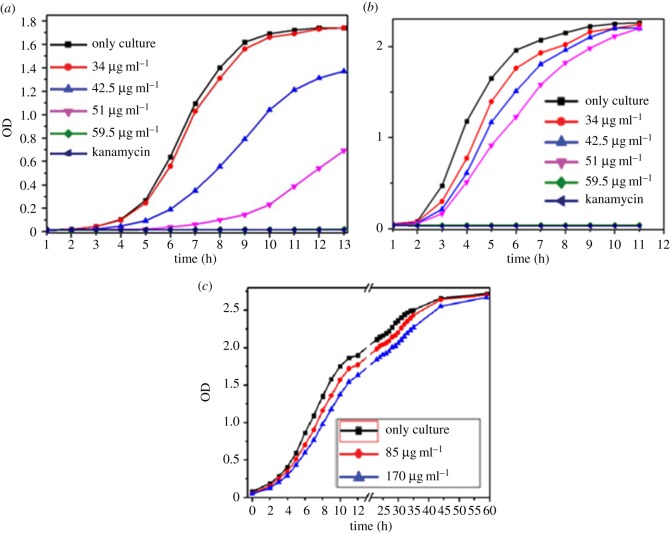
(*a*) Growth curve of *Bacillus subtilis* against compound **1** and kanamycin as the reference showing that the growth significantly decreases. (*b*) Growth curve of *Vibrio cholerae* against compound **1** and kanamycin as the reference exhibiting the growth of *Vibrio cholerae* gradually decreases with increasing compound concentration*.* (*c*) Growth curve of yeast against compound **1** and kanamycin as the reference showing the growth of yeast is not inhibited significantly by compound **1** even at a very high concentration.

Inspired by this result, we tried to check the antibacterial effect of the compound quantitatively. The strength of an antibacterial agent is expressed in terms of the minimum inhibitory concentration (MIC). The results indicate that as the compound concentration increases, bacterial growth is gradually inhibited, which is reflected from the gradual lowering of the OD (optical density) for each set, and at a compound concentration of 85 µg ml^−1^ the bacterial growth becomes fully diminished like that of kanamycin (electronic supplementary material, figure 3).

**Figure 3.**
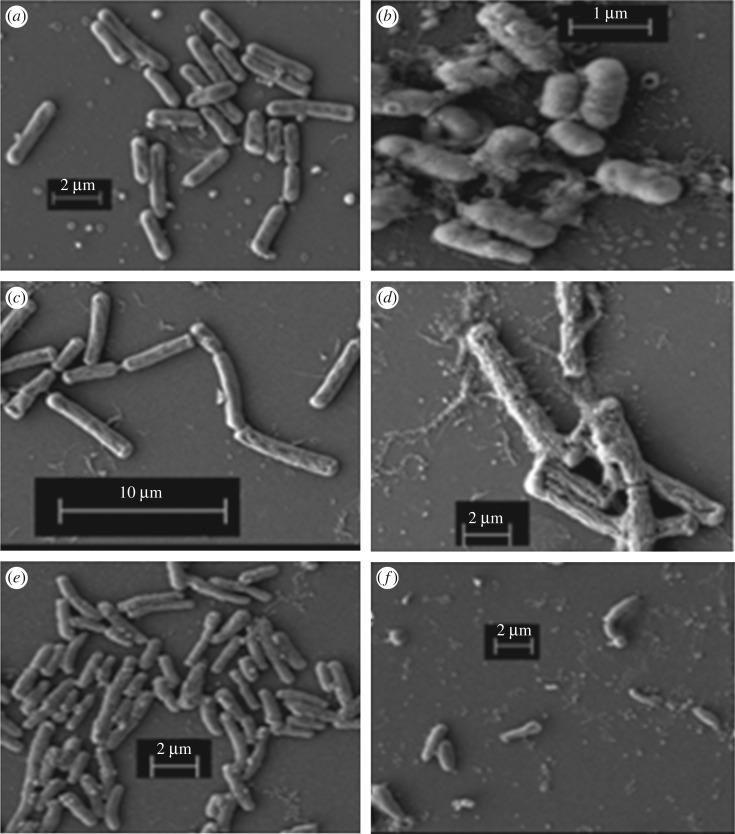
(*a*) FE-SEM image of *E. coli*, (*b*) FE-SEM image of compound **1**-treated *E. coli*. (*c*) FE-SEM image of *Bacillus subtilis*, (*d*) FE-SEM image of compound **1**-treated *Bacillus subtilis*. (*e*) FE-SEM image of *Vibrio cholerae*, (*f*) FE-SEM image of compound **1**-treated *Vibrio cholerae*.

We have also investigated the effect of compound **1** on other species of bacteria like *Bacillus subtilis.* The experimental data show that the MIC of the amino acid for *Bacillus subtilis* is 59.5 µg ml^−1^ (figure 2*a*). Further, we are interested to know whether compound **1** is active on pathogenic bacteria like *Vibrio cholerae* or not. The results show that with increase in compound **1** concentration the growth of *Vibrio cholerae* significantly decreases and the MIC value is 59.5 µg ml^−1^ (figure 2*b*). For a potential antibiotic, the compound should be active only on bacteria, and should not have a harmful effect on eukaryotes. The result (figure 2*c*) indicates that the growth of the yeast is not inhibited significantly by compound **1** even at very high concentration.

To study the morphological changes of bacterial shape after treatment with compound **1**, FE-SEM experiments were performed. FE-SEM images (figure 3) show the images of healthy bacterial cells in the control experiment. However, upon treating the bacteria with compound **1**, the *E. coli, Bacillus subtilis* and *Vibrio cholerae* bacterial cells become completely deformed. These results reveal that compound **1** significantly affects the bacteria.

**Figure 4.**
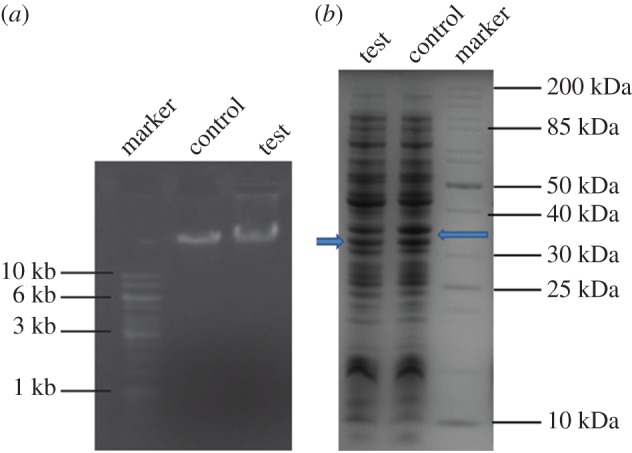
(*a*) Gel electrophoresis experiment for extracted bacterial DNA. (*b*) Gel electrophoresis experiment for extracted bacterial protein showing that the intensity of the protein band marked by the arrow has reduced in the presence of compound **1**.

Moreover, to study the effect of compound **1** on bacteria, *in vitro* experiments were carried out. For the *in vitro* experiments, only *E. coli* bacteria was used. The interactions of DNA with other chemical and biological functionalities lead to a diversity of binding and reaction patterns [26,27]. Previously, peptide-based molecules as well as iron-containing complexes have been reported as DNA-binding agents [28,29]; however, even some of them have been reported to damage the DNA [30]. Inspired from the above reports, we wanted to study whether compound **1** has the ability to damage or degrade DNA. For that purpose, we have isolated the bacterial genome from the control experiment and compound **1**-treated bacterial cell by the standard procedure and compared them. Then gel electrophoresis was performed with the extracted DNA materials. Figure 4*a* indicates that no extra DNA band appears for the compound **1**-treated bacterial DNA. In both the test sample and the control the band positions are the same. This result indicates that no bacteria DNA degradation occurs by treatment with 1-(2-aminophenyl)-1H-1,2,3-triazole-4-carboxylic acid.

Many drug molecules have been known to bind with protein and make drug–protein adducts [31]. Bacterial cell division can be inhibited by targeting the specific protein [32]. Now, to investigate whether 1-(2-aminophenyl)-1H-1,2,3-triazole-4-carboxylic acid can bind or damage some bacterial protein or not, we need to isolate the bacterial protein from the control experiment and compound **1**-treated bacteria and compare them. The proteins were isolated from the bacterial cell using the standard procedure. The results (figure 4*b*) indicate that the intensity of the protein band marked by the arrow are going to be reduced in the compound **1-**treated sample compared to the control one, while the intensity of the other bands remain the same in both samples. Hence, compound **1** significantly interacts with some bacterial proteins and degrades them, causing bacterial death.

## 4. Conclusion

In conclusion, we have designed and synthesized a new ε-amino acid, namely 1-(2-aminophenyl)-1H-1,2,3-triazole-4-carboxylic acid by click reaction. From X-ray crystallography, the amino acid adopts a kink-like structure where the phenyl and triazole rings are perpendicular to each other and the amine and acid groups maintain an angle of 60°. The amino acid has significant antibacterial activity. The MIC value of the compound for *Bacillus subtilis* and *Vibrio cholerae* is 59.5 µg ml^−1^. However, the amino acid does not have any harmful effect on eukaryotes. The activity of the amino acid on bacteria was further studied by DNA and protein binding and degradation study, dye-binding assay and morphological analysis, which show that the amino acid significantly interacts with bacterial proteins and degrades them, causing bacterial death. The results presented here show that the synthetic amino acid may eventually be effective against pathogens, but additional studies are required to reach this goal.

## Supplementary Material

1-(2-Aminophenyl)-3H-[1,2,3] triazole-4-carboxylic acid: activity against Gram-positive and Gram-negative pathogens including Vibrio Cholerae

## Data accessibility

The datasets supporting this article have been uploaded as part of the electronic supplementary material.

## References

[1] Taubes G 2008 The bacteria fight back. Science 321, 356–361. (doi:10.1126/science.321.5887.356) 18635788 10.1126/science.321.5887.356

[2] Zasloff M 2002 Antimicrobial peptides of multicellular organisms. Nature 415, 389–395. (doi:10.1038/415389a) 11807545 10.1038/415389a

[3] Maianti JP, Hanessian S 2016 Structural hybridization of three aminoglycoside antibiotics yields a potent broad-spectrum bactericide that eludes bacterial resistance enzymes. Med. Chem. Commun. 7, 170–176. (doi:10.1039/C5MD00429B)

[4] Romero D, Traxler MF, López D, Kolter R 2011 Antibiotics as signal molecules. Chem. Rev. 111, 5492–5505. (doi:10.1021/cr2000509) 21786783 10.1021/cr2000509 PMC3173521

[5] Hancock REW, Sahl HG 2006 Antimicrobial and host-defense peptides as new antiinfective therapeutic strategies. Nat. Biotechnol. 24, 1551–1557. (doi:10.1038/nbt1267) 17160061 10.1038/nbt1267

[6] Andersson DI, Hughes D, Kubicek-Sutherland JZ 2016 Mechanisms and consequences of bacterial resistance to antimicrobial peptides*.* Drug Resist. Updat. 26, 43–57. (doi:10.1016/j.drup.2016.04.002) 27180309 10.1016/j.drup.2016.04.002

[7] Zhang L, Gallo RL 2016 Antimicrobial peptides. Curr. Biol. 26, R14–R19. (doi:10.1016/j.cub.2015.11.017) 26766224 10.1016/j.cub.2015.11.017

[8] Zhao C, Liu L, Lehrer RI 1994 Identification of a new member of the protegrin family by cDNA cloning. FEBS Lett. 346, 285–288. (doi:10.1016/0014-5793(94)00493-5) 8013647 10.1016/0014-5793(94)00493-5

[9] Miyata T, Tokunaga F, Yoneya T, Yoshikawa K, Iwanaga S, Niwa M, Takao T, Shimonishi YJ 1989 Antimicrobial peptides isolated from horseshoe crab hemocytes, tachyplesin II, and polyphemusins I and II: chemical structures and biological activity. J. Biochem. 106, 663–668. (doi:10.1093/oxfordjournals.jbchem.a122913) 2514185 10.1093/oxfordjournals.jbchem.a122913

[10] Garcia AE, Osapay G, Tran PA, Yuan J, Selsted ME 2008 Isolation, synthesis, and antimicrobial activities of naturally occurring θ-defensin isoforms from baboon leukocytes. Infect. Immun. 76, 5883–5891. (doi:10.1128/IAI.01100-08) 18852242 10.1128/IAI.01100-08 PMC2583559

[11] Srinivas N et al. 2010 Peptidomimetic antibiotics target outer-membrane biogenesis in *Pseudomonas aeruginosa*. Science 327, 1010–1013. (doi:10.1126/science.1182749) 20167788 10.1126/science.1182749

[12] Wang CK, King GJ, Conibear AC, Ramos MC, Chaousis SS, Henriques T, Craik DJ 2012 Synthetic mimics of antimicrobial peptides with immunomodulatory responses. J. Am. Chem. Soc. 134, 11 088–11 091. (doi:10.1021/ja303304j) 22697149 10.1021/ja303304j PMC3406751

[13] Li F, Collins JG, Keene FR 2015 Ruthenium complexes as antimicrobial agents. Chem. Soc. Rev. 44, 2529–2542. (doi:10.1039/C4CS00343H) 25724019 10.1039/c4cs00343h

[14] Irwansyah I, Li YQ, Shi W, Qi D, Leow WR, Tang MB, Li S, Chen X 2015 Gram-positive antimicrobial activity of amino acid-based hydrogels. Adv. Mater. 27, 648–654. (doi:10.1002/adma.201403339) 25447243 10.1002/adma.201403339

[15] Funt LD, Tomashenko OA, Khlebnikov AF, Novikov MS, Ivanov AY 2016 Synthesis, transformations of pyrrole- and 1,2,4-triazole-containing ensembles, and generation of pyrrole-substituted triazole NHC. J. Org. Chem. 81, 11 210–11 221. (doi:10.1021/acs.joc.6b02200) 10.1021/acs.joc.6b02200 27726365

[16] Martins P, Jesus J, Santos S, Raposo LR, Roma-Rodrigues C, Baptista PV, Fernandes AR 2015 Heterocyclic anticancer compounds: recent advances and the paradigm shift towards the use of nanomedicine's tool box. Molecules 20, 16 852–16 891. (doi:10.3390/molecules200916852) 10.3390/molecules200916852 PMC6331900 26389876

[17] Somu RV, Boshoff H, Qiao C, Bennett EM, Barry CE, Aldrich CC 2006 Rationally designed nucleoside antibiotics that inhibit siderophore biosynthesis of *Mycobacterium tuberculosis*. J. Med. Chem. 49, 31–34. (doi:10.1021/jm051060o) 16392788 10.1021/jm051060o

[18] Boechat N, Ferreira ML, Pinheiro LC, Jesus AM, Leite MM, Júnior CC, Aguiar AC, de Andrade IM, Krettli AU 2014 New compounds hybrids 1*H*-1,2,3-triazole-quinoline against *Plasmodium falciparum*. Chem. Biol. Drug Des. 84, 325–332. (doi:10.1111/cbdd.12321) 24803084 10.1111/cbdd.12321

[19] Jiang B, Huang X, Yao H, Jiang J, Wu X, Jiang S, Wang Q, Lu T, Xu J 2014 Discovery of potential anti-inflammatory drugs: diaryl-1,2,4-triazoles bearing *N*-hydroxyurea moiety as dual inhibitors of cyclooxygenase-2 and 5-lipoxygenase. Org. Biomol. Chem. 12, 2114–2127. (doi:10.1039/c3ob41936c) 24562695 10.1039/c3ob41936c

[20] Gupta D, Jain DK 2015 Synthesis, antifungal and antibacterial activity of novel 1,2,4-triazole derivatives. J. Adv. Pharm. Technol. Res. 6, 141–146. (doi:10.4103/2231-4040.161515) 26317080 10.4103/2231-4040.161515 PMC4542402

[21] Maji K, Sarkar R, Bera S, Haldar D 2014 A small molecule peptidomimetic of spider silk and webs. Chem. Commun. 50, 12 749–12 752. (doi:10.1039/C4CC04475D) 10.1039/c4cc04475d 25204650

[22] Maity S, Kumar R, Maity SK, Jana P, Bera S, Haldar D 2013 Synthesis and study of 2-acetyl amino-3-[4-(2-amino-5-sulfo-phenylazo)-phenyl]-propionic acid: a new class of inhibitor for hen egg white lysozyme amyloidogenesis. Med. Chem. Commun. 4, 530–536. (doi:10.1039/c2md20236k)

[23] Kolb HC, Finn MG, Sharpless KB 2001 Click chemistry: diverse chemical function from a few good reactions. Angew. Chem. Int. Ed. 40, 2004–2021. (doi:10.1002/1521-3773(20010601)40:11<2004::AID-ANIE2004>3.0.CO;2-5) 10.1002/1521-3773(20010601)40:11<2004::AID-ANIE2004>3.0.CO;2-5 11433435

[24] Kamal A, Hussaini SMA, Sucharitha ML, Poornachandra Y, Sultana F, Kumara CG 2015 Synthesis and antimicrobial potential of nitrofuran–triazole congeners. Org. Biomol. Chem. 13, 9388–9397. (doi:10.1039/C5OB01353D) 26238045 10.1039/c5ob01353d

[25] Rahelivao MP, Lübken T, Gruner M, Kataeva O, Ralambondrahety R, Andriamanantoanina H, Checinski MP, Bauer I, Knölker H-J 2017 Isolation and structure elucidation of natural products of three soft corals and a sponge from the coast of Madagascar. Org. Biomol. Chem. 15, 2593–2608. (doi:10.1039/C7OB00191F) 28267183 10.1039/c7ob00191f

[26] Krishnan Y, Simmel FC 2011 Nucleic acid based molecular devices. Angew. Chem. Int. Ed. 50, 3124–3156. (doi:10.1002/anie.200907223) 10.1002/anie.200907223 21432950

[27] George NP, Keck JL 2009 Slip sliding on DNA. Nature 461, 1067–1068. (doi:10.1038/4611067a) 19847254 10.1038/4611067a PMC2819181

[28] Vázquez ME, Caamaño AM, Mascareñas JL 2003 From transcription factors to designed sequence-specific DNA-binding peptides. Chem. Soc. Rev. 32, 338–349. (doi:10.1039/B206274G) 14671789 10.1039/b206274g

[29] Loganathan R, Ramakrishnan Suresh S, Riyasdeen EA, Akbarsha MA, Palaniandavar M 2011 DNA binding, prominent DNA cleavage and efficient anticancer activities of Tris(diimine)iron(II) complexes. Dalton Trans. 40, 3524–3536. (doi:10.1039/c0dt00466a) 21369607 10.1039/c0dt00466a

[30] Midorikawa K, Murata M, Oikawa S, Oikawa S, Saeko T-O, Kawanishi S 2000 DNA damage by dimethylformamide: role of hydrogen peroxide generated during degradation. Chem. Res. Toxicol. 13, 309–315. (doi:10.1021/tx990139r) 10775332 10.1021/tx990139r

[31] Tailor A, Waddington JC, Meng X, Park BK 2016 Detection of drug bioactivation in vivo: mechanism of nevirapine–albumin conjugate formation in patients. Chem. Res. Toxicol. 29, 1912–1935. (doi:10.1021/acs.chemrestox.6b00147) 23448204 10.1021/tx4000107

[32] Avila LBR et al. 2013 Synthetic inhibitors of bacterial cell division targeting the GTP-binding site of FtsZ. ACS Chem. Biol. 8, 2072–2083. (doi:10.1021/cb400208z) 23855511 10.1021/cb400208z

