# Supplementary material for: 1-(2-aminophenyl)-1H-1,2,3-triazole-4-carboxylic acid: activity against Gram-positive and Gram-negative pathogens including Vibrio cholerae
Source: R Soc Open Sci. 2017 Oct 18;4(10):170684. doi: 10.1098/rsos.170684 (PMC5666259; doi:10.1098/rsos.170684)
Supplement: 1-(2-Aminophenyl)-3H-[1,2,3] triazole-4-carboxylic acid: activity against Gram-positive and Gram-negative pathogens including Vibrio Cholerae [file rsos170684supp1.doc]

**Supporting information**

**1-(2-aminophenyl)-1H-1,2,3-triazole-4-carboxylic acid: activity against Gram-positive and Gram-negative pathogens including *Vibrio cholerae***

Krishnendu Maji and Debasish Haldar*

Department of Chemical Sciences, Indian Institute of Science Education and Research Kolkata, Mohanpur, West Bengal 741246, India, E-mail: deba_h76@yahoo.com; deba_h76@iiserkol.ac.in

Table of contents

| ESI Figure S1 | 2 | Figure S3 | 5 |
| --- | --- | --- | --- |
| ESI Figure S2 | 2 | Figure S4 | 5 |
| ESI Figure S3 | 2 | Figure S5 | 5 |
| Scheme 1 | 3 | Figure S6 | 6 |
| Scheme 2 | 3 | Figure S7 | 6 |
| Scheme 3 | 3 | Figure S8 | 7 |
| Scheme 4 | 3 | Figure S9 | 7 |
| Scheme 5 | 3 | Figure S10 | 8 |
| Figure S1 | 4 | Figure S11 | 8 |
| Figure S2 | 4 |  |  |


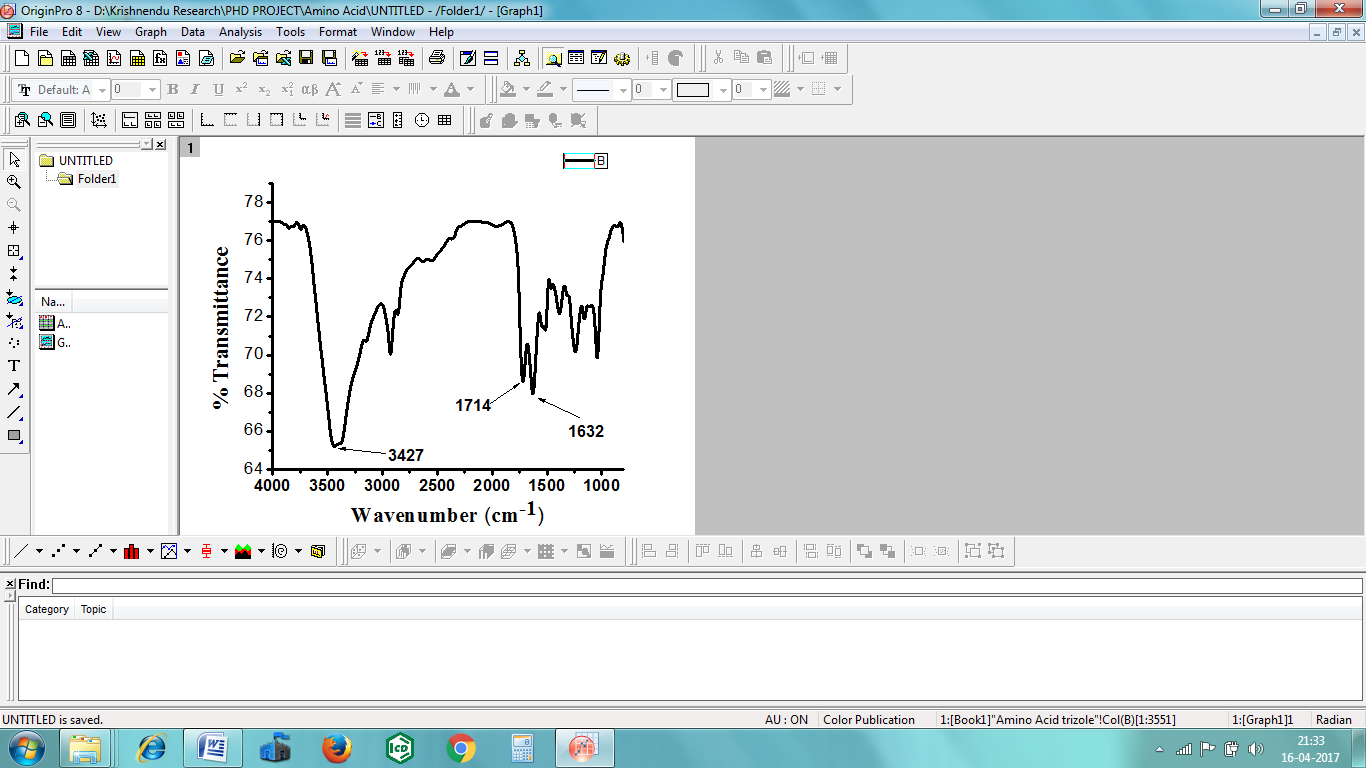


**ESI Figure S1**. Solid state FT-IR spectra of Compound 1-(2-aminophenyl)-1H-1,2,3-triazole-4-carboxylic acid.


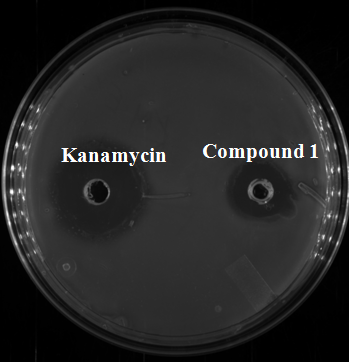

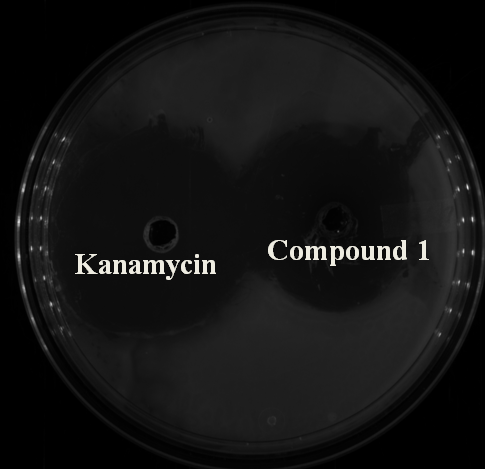


a b

**ESI Figure S2.** The growth inhibition zone of (a) *E. coli* and (b) *Bacillus subtilis* indicates the significant antibacterial activity of compound **1**.


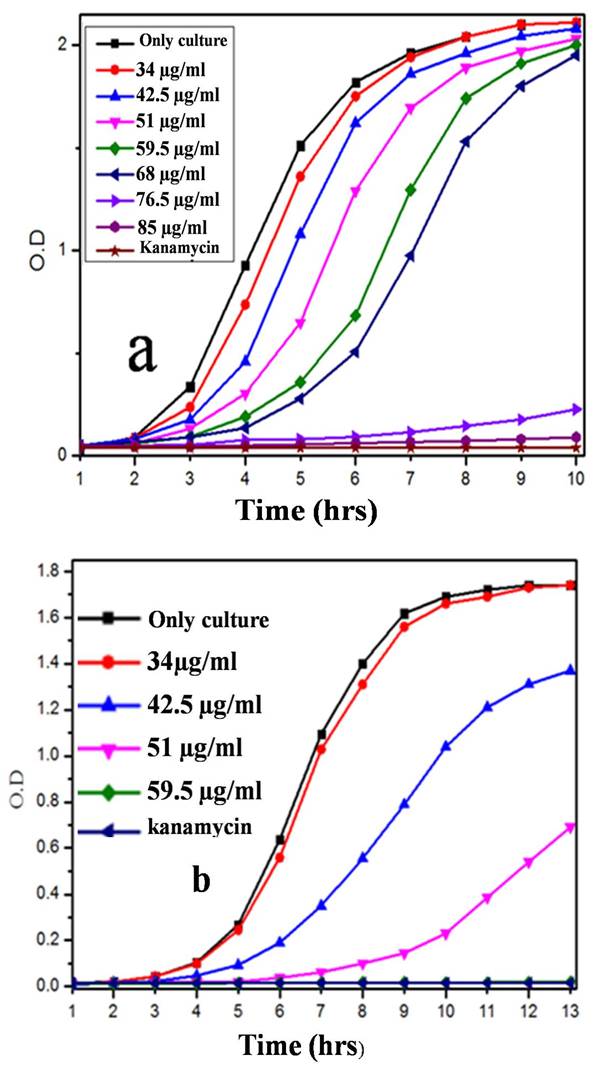


**ESI Figure S3.** Growth Curve of *E. coli* indicates that with gradual increase in concentration of compound 1, bacterial growth become inhibited.

**Scheme1:** Synthesis of 2-Pthalimidonitrobenzene

**Scheme2:** Synthesis of 2-Phthalimidoaniline

**Scheme 3:** Synthesis of 2-Phthalimidophenyl Azide

**Scheme 4:** Synthesis of 2-aminophenyl azide

**Scheme 5:** Synthesis of 1-(2-aminophenyl)-1H-1,2,3-triazole-4-carboxylic acid

**
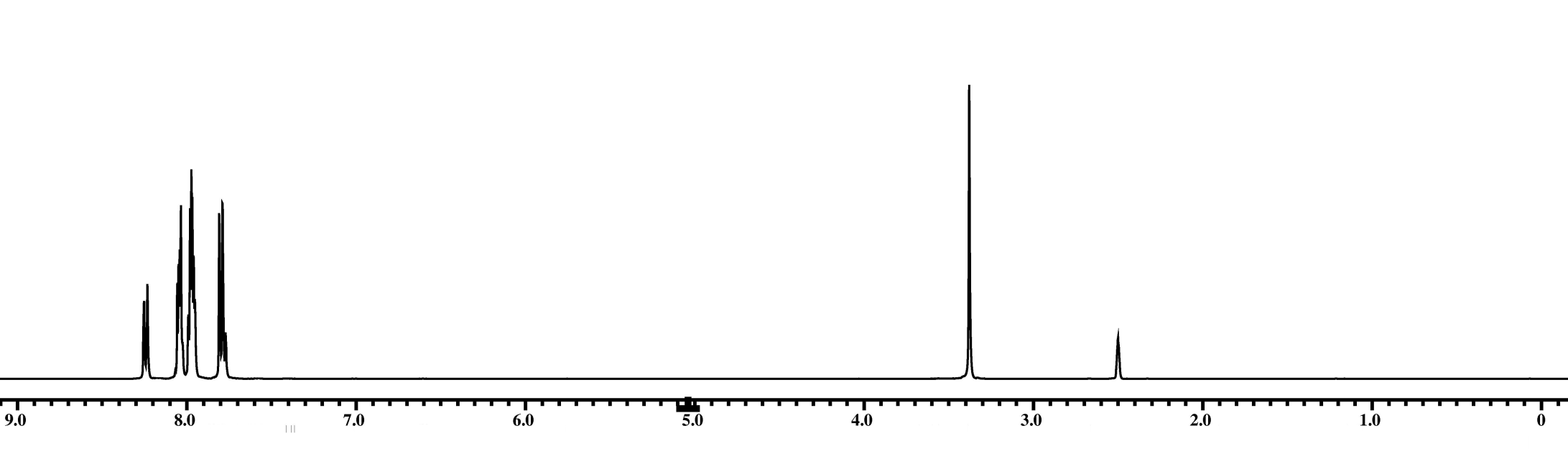
**

**Figure S1:** ^1^H NMR (400MHz, DMSO-*d_6_*, δ in ppm) spectrum of 2-Pthalimidonitrobenzene.


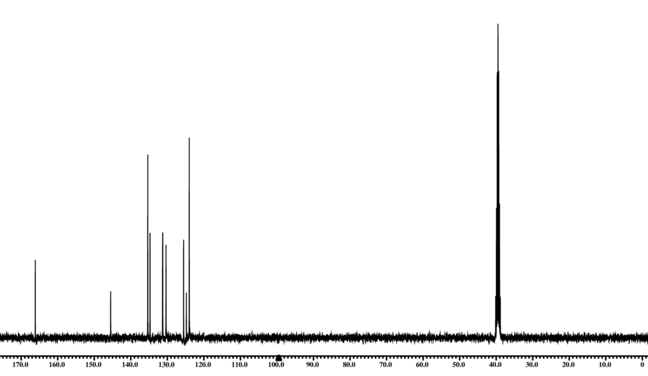


**Figure S2:** ^13^C NMR (100MHz, DMSO-*d_6_*, δ in ppm) spectrum of 2-Pthalimidonitrobenzene.


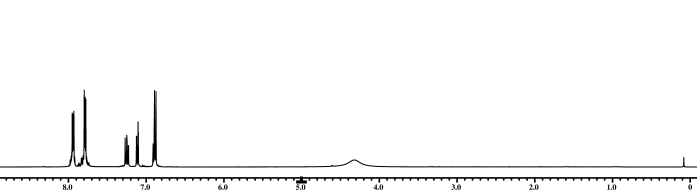


**Figure S3:**^. 1^H NMR (400MHz, CDCl_3_, δ in ppm) spectrum of 2-Phthalimidoaniline.


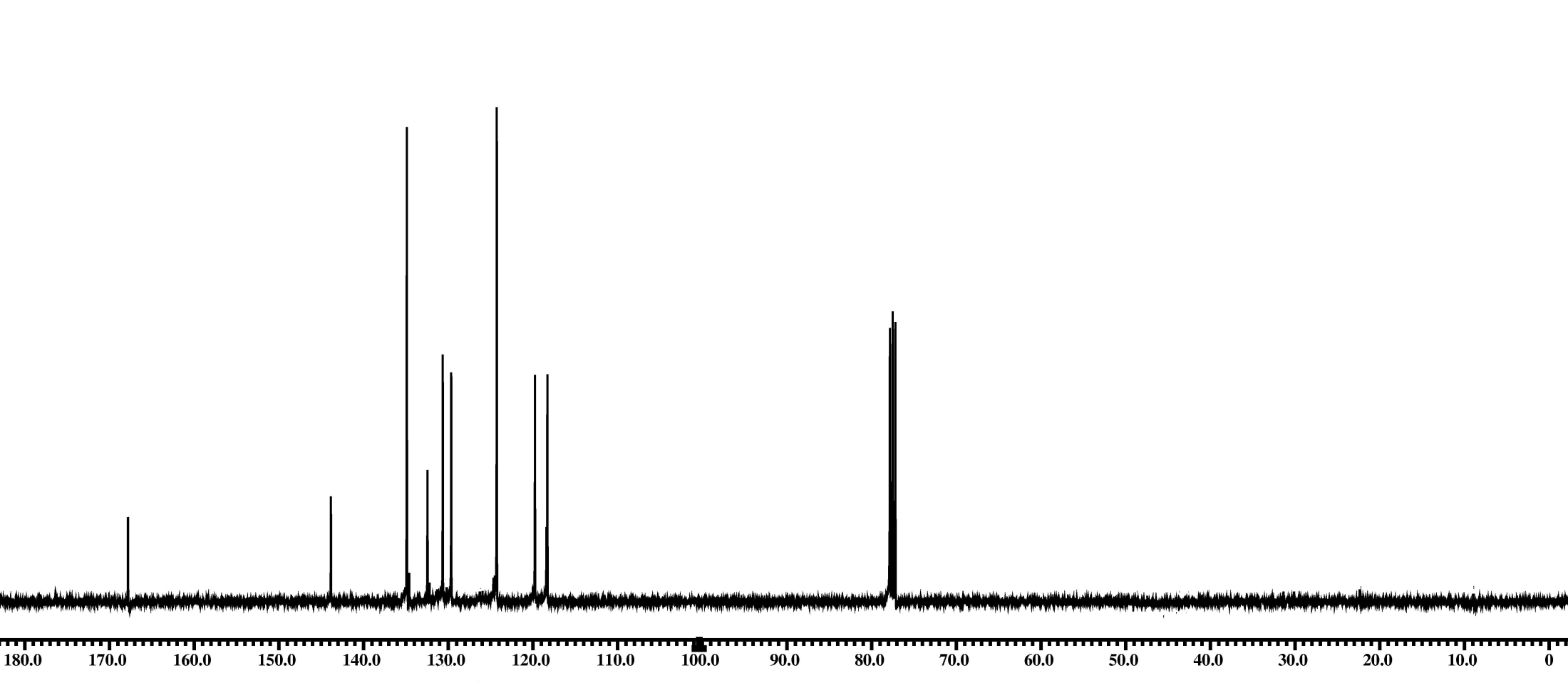


**Figure S4:** ^13^CNMR (100MHz, CDCl_3_, δ in ppm) spectrum of 2-Phthalimidoaniline.


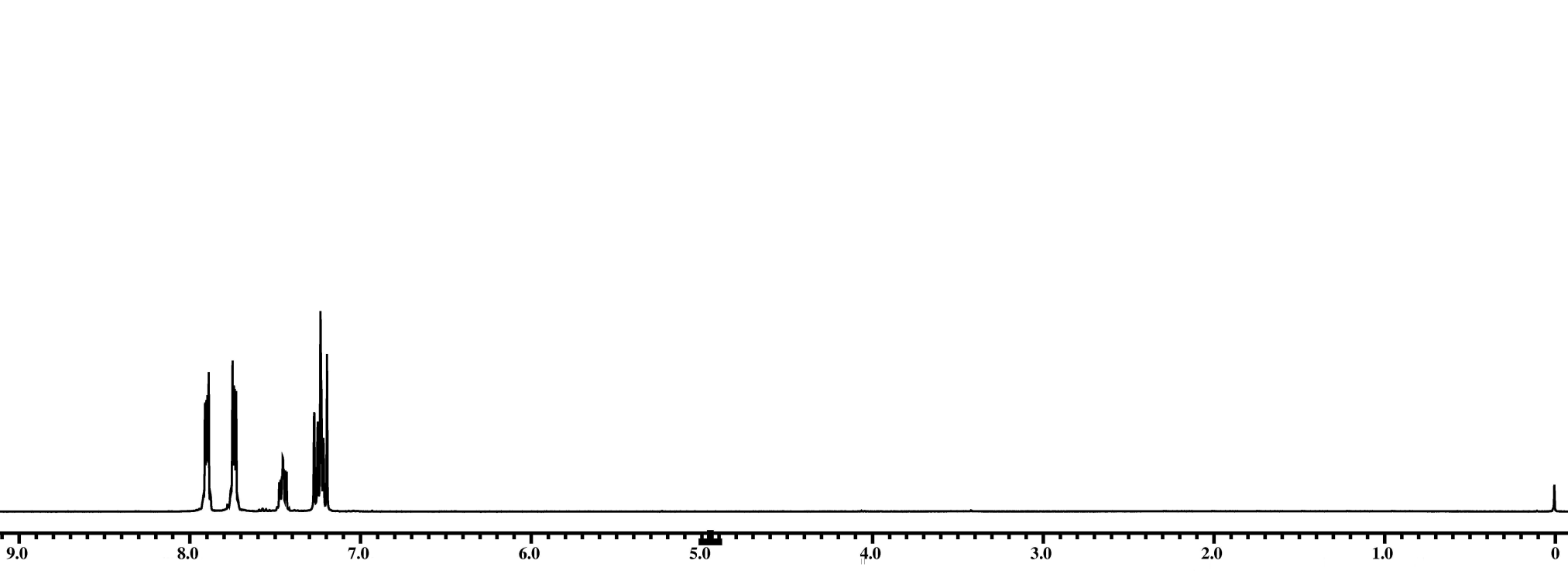


**Figure S5:** ^1^H NMR (400MHz, CDCl_3_, δ in ppm) spectrum of 2-Phthalimidophenyl Azide.


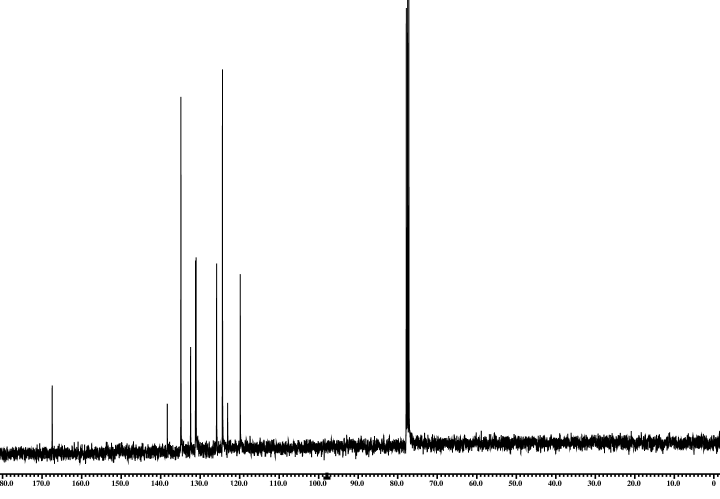


**Figure S6:** ^13^CNMR (100MHz, CDCl_3_, δ in ppm) spectrum of 2-Phthalimidophenyl Azide.


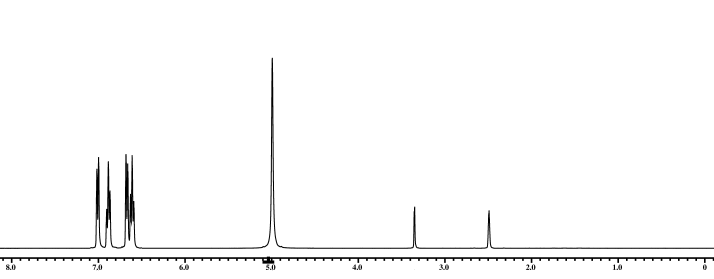


**Figure S7:**^. 1^H NMR (400MHz, DMSO-*d_6_*, δ in ppm) spectrum of 2-Azidoaniline.


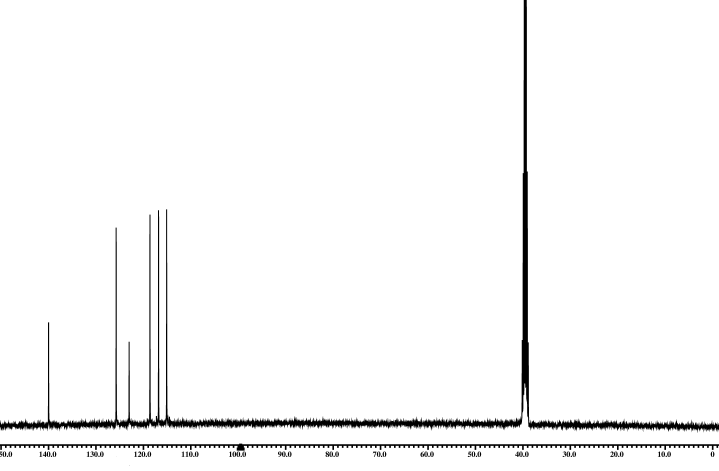


**Figure S8:**^.13^CNMR (100MHz, DMSO-*d_6_*, δ in ppm) spectrum of 2-Azidoaniline.


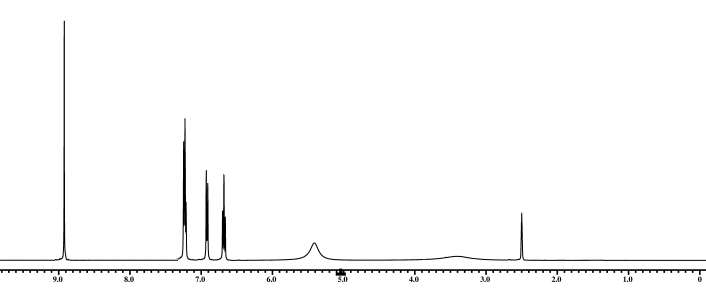


**Figure S9:**.^1^HNMR (400MHz, DMSO-*d_6_*, δ in ppm) spectrum of 1-(2-aminophenyl)-1H-1,2,3-triazole-4-carboxylic acid.


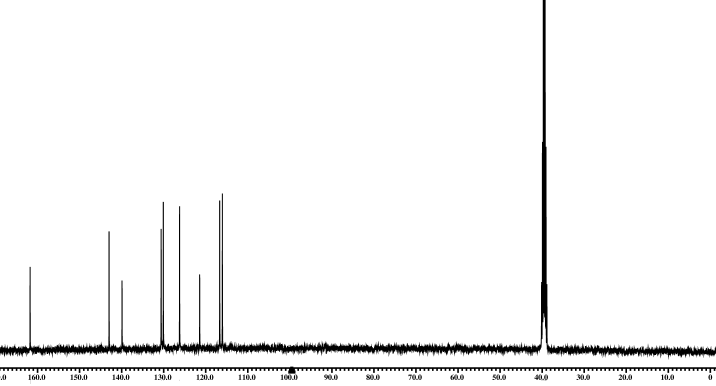


**Figure S10:** ^13^C NMR (100MHz, DMSO-*d_6_*, δ in ppm) spectrum of 1-(2-aminophenyl)-1H-1,2,3-triazole-4-carboxylic acid.

**
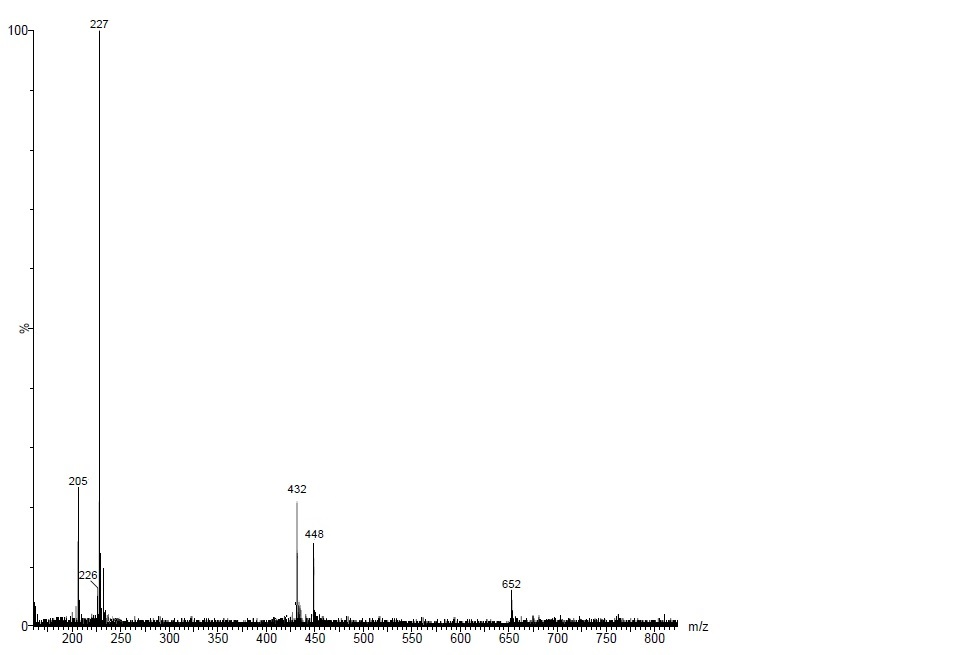
**

**Figure S11:** Mass spectrum of Compound 1-(2-aminophenyl)-1H-1,2,3-triazole-4-carboxylic acid.
